# Impact of Different Glomerular Filtration Rate Equations on Metformin Eligibility in Patients with Diabetes Mellitus and Chronic Kidney Disease

**DOI:** 10.3390/jcm15072493

**Published:** 2026-03-24

**Authors:** Sirinapa Traiwanatham, Methus Jantarapootirat, Kanin Thammavaranucupt, Supawadee Suppadungsuk, Chutintorn Sriphrapradang

**Affiliations:** 1 Faculty of Medicine Ramathibodi Hospital, Ramathibodi School of Medicine, Chakri Naruebodindra Medical Institute, Mahidol University, Samut Prakan 10540, Thailand; sirinapa.tra@mahidol.ac.th (S.T.); methus.jan@mahidol.ac.th (M.J.); kanin.tha@mahidol.ac.th (K.T.); supawadee.sup@mahidol.ac.th (S.S.); 2 Department of Medicine, Faculty of Medicine Ramathibodi Hospital, Mahidol University, Bangkok 10400, Thailand

**Keywords:** biguanide, chronic kidney failure, chronic renal insufficiency, kidney function, metformin, diabetes mellitus

## Abstract

**Background:** Chronic kidney disease (CKD) affects up to 40% of patients with diabetes mellitus and has important implications for metformin safety. Although estimated glomerular filtration rate (eGFR)-based dosing is recommended, there is no consensus on the optimal estimating equation, which may lead to inconsistent treatment decisions. **Methods:** This retrospective study analyzed 46,788 Thai patients with diabetes from 2014 to 2024. eGFR was calculated using five equations, and CKD stages and metformin eligibility were evaluated according to U.S. FDA and KDIGO guidelines. **Results:** Median eGFR differed significantly across equations (*p*-value < 0.001), with the highest values observed using CKD-EPI 2009 and the lowest using Cockcroft–Gault. Among 30,805 metformin users, 0.6–3.7% had eGFR < 30 mL/min/1.73 m^2^ depending on the equation used. Agreement with CKD-EPI 2009 ranged from 96 to 99 than 1% (≈3% with Cockcroft–Gault) were unnecessarily excluded from metformin therapy. CKD-EPI 2021 yielded approximately 4.5 mL/min/1.73 m^2^ higher eGFR values, reclassifying 19% of patients to a better CKD stage. **Conclusions:** Differences among eGFR equations affect CKD staging and metformin eligibility. CKD-EPI 2009, CKD-EPI 2021, S-MDRD, and Thai GFR showed good agreement, whereas Cockcroft–Gault may underestimate renal function, potentially excluding patients who could safely continue metformin. Until outcome data are available, the widely used CKD-EPI equation remains the preferred reference due to its consistency with other standard equations. Further prospective studies are needed to evaluate the clinical impact of equation choice on metformin management.

## 1. Introduction

Chronic kidney disease (CKD) is a leading long-term microvascular complication of diabetes mellitus (DM), affecting approximately 30–40% of patients and representing the most common cause of CKD and end-stage renal disease worldwide [1,2]. Renal function plays a critical role in determining the safety of many medications, particularly metformin. Metformin is not metabolized, does not bind to plasma proteins, and is excreted unchanged via active tubular secretion in the proximal tubules [3]. Consequently, impaired renal function may lead to drug accumulation and increased risk of metformin-associated lactic acidosis [4].

The U.S. Food and Drug Administration (FDA) recommends against the use of metformin in patients with an estimated glomerular filtration rate (eGFR) < 30 mL/min/1.73 m^2^ and advises caution when initiating treatment in those with an eGFR 30–45 mL/min/1.73 m^2^. In patients whose eGFR declines to <45 mL/min/1.73 m^2^ during ongoing treatment, reassessment of the risk–benefit profile is recommended, and the maximum daily dose should be limited to 1000 mg [5]. Although current guidelines support eGFR-based dosing of metformin, they do not specify which estimating equation should be applied. This contrasts with non-vitamin K oral anticoagulants (NOACs), for which the Cockcroft–Gault equation is consistently recommended to estimate creatinine clearance [6,7]. This lack of equation-specific guidance for metformin may lead to inconsistencies in clinical practice and highlights the need for standardized recommendations.

Commonly used eGFR equations include the Chronic Kidney Disease Epidemiology Collaboration (CKD-EPI) equation 2009 and 2021, the Standard Modification of Diet in Renal Disease (S-MDRD) equation, and the Cockcroft–Gault formula [8]. The updated 2021 CKD-EPI creatinine equation was refitted from the 2009 CKD-EPI equation and excludes race, incorporating serum creatinine, age, and sex as variables. Removal of race-based adjustment was intended to minimize potential bias and enhance equity in renal function assessment across populations. In addition, a population-specific Thai GFR equation has been developed [9]. Because these equations differ in accuracy, they may yield discrepant eGFR estimates and lead to misclassification of renal function [10], which could subsequently influence metformin eligibility and dose adjustment. Therefore, this study aimed to compare five eGFR equations in Thai patients with DM and assess their impact on metformin prescribing eligibility and dosing decisions.

## 2. Materials and Methods

### 2.1. Study Design and Participants

This retrospective study was conducted at two university-affiliated medical school hospitals in Thailand. Eligible participants were identified from electronic medical records between 1 November 2014 and 31 October 2024 using relevant International Classification of Diseases, 10th Revision (ICD-10) codes. The study was approved by the institutional review boards. Owing to the retrospective design and use of de-identified data, the requirement for informed consent was waived.

Participants were included if they had a diagnosis of DM, identified by ICD-10 codes E10 (type 1 DM), E11 (type 2 DM), E12 (malnutrition-related DM), E13 (other specified DM), and E14 (unspecified DM).

**Figure 1:**
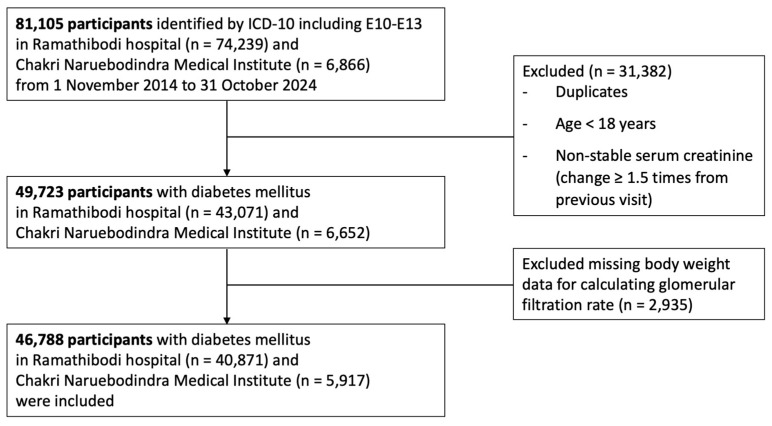
Flow diagram for study participants.

Additional inclusion criteria were age ≥ 18 years and availability of at least two serum creatinine measurements taken at least 3 months apart. To ensure stable kidney function, the two creatinine values were required to differ by less than 1.5-fold. A total of 46,788 patients met the eligibility criteria and were included in the final analysis (Figure 1).

### 2.2. eGFR Equations

The most recent serum creatinine (Scr) value (mg/dL), along with age, sex, and body weight, was used to calculate eGFR (mL/min/1.73 m^2^) using five different equations:

Chronic Kidney Disease–Epidemiology Collaboration (CKD-EPI) 2021 [11]

eGFR = 142 × min (Scr/κ,1)^α^ × max (Scr/κ,1)^−1.200^ × 0.9938^Age^ × [1.012 if female]

where κ = 0.7 for females and 0.9 for males, α = −0.241 for females and −0.302 for males. The function min indicates the minimum of Scr/Ƙ or 1, and max indicates the maximum of sCr/Ƙ or 1.

Chronic Kidney Disease–Epidemiology Collaboration (CKD-EPI) 2009 [12]

eGFR = 141 × min(Scr/κ,1)^α^ × max(Scr/κ,1)^−1.209^ × 0.993^Age^ × [1.018 if female] × [1.159 if Black]

where κ = 0.7 for females and 0.9 for males, and α = −0.329 for females and −0.411 for males. The function min indicates the minimum of Scr/Ƙ or 1, and max indicates the maximum of Scr/κ or 1.

Standard Modification of Diet in Renal Disease (S-MDRD) [13]

eGFR = 175 × Scr^−1.154^ × Age^−0.203^ × [0.742 if female]

Thai GFR [9]

eGFR = 375.5 × Scr^−0.848^ × Age^−0.364^ × [0.712 if female]

Cockcroft–Gault [14]

Creatinine Clearance (CrCl) = (140 − Age) × (Weight) × [0.085 if female]/(72 × Scr)

CrCl derived from the Cockcroft–Gault equation was converted to mL/min/1.73 m^2^ for comparison by adjusting for body surface area using the DuBois formula.

The CKD-EPI 2009 equation was selected as the reference equation because the study period (2014–2024) largely reflects clinical practice prior to the widespread implementation of the CKD-EPI 2021 equation. In addition, cystatin C was not routinely available in our setting, precluding the use of the creatinine–cystatin C equation.

### 2.3. CKD Staging

CKD was classified by eGFR categories based on the Kidney Disease: Improving Global Outcomes (KDIGO) 2024 guidelines as follows: G1 (≥90 mL/min/1.73 m^2^), G2 (60–89 mL/min/1.73 m^2^), G3a (45–59 mL/min/1.73 m^2^), G3b (30–44 mL/min/1.73 m^2^), G4 (15–29 mL/min/1.73 m^2^), and G5 (<15 mL/min/1.73 m^2^) [15].

### 2.4. Metformin Dose Adjustment Recommendations

In 2016, the U.S. Food and Drug Administration (FDA) revised its recommendations for metformin use, replacing serum creatinine–based assessment with eGFR-based evaluation of kidney function. According to FDA guidance, metformin use was categorized as follows: (1) contraindicated if eGFR < 30 mL/min/1.73 m^2^; (2) not recommended for initiation if eGFR < 45 mL/min/1.73 m^2^; (3) reassessment of the risk-benefit profile advised if eGFR declines to <45 mL/min/1.73 m^2^ during ongoing treatment; (4) periodic monitoring of renal function required; and (5) temporary discontinuation recommended prior to iodinated contrast imaging in patients with eGFR 30–60 mL/min/1.73 m^2^ [16]. However, the FDA does not specify a preferred eGFR estimating equation for guiding metformin eligibility or dose adjustment in patients with impaired kidney function.

### 2.5. Agreement

Agreement was defined as concordance between each estimating equation and the reference equation, the CKD-EPI 2009 equation, in recommending metformin eligibility and dosing category.

Undertreatment was operationally defined as when an equation recommended discontinuation of metformin or a lower dose than recommended by the reference equation, whereas overtreatment was defined as recommending metformin use or a higher dose when the reference equation would not. These definitions were used to describe differences in classification and do not imply that the reference equation is the most accurate method for estimating kidney function.

### 2.6. Statistical Analyses

Descriptive statistics was used to summarize baseline characteristics, including sex, age, body weight, Scr, and metformin use. Continuous variables were presented as mean ± standard deviation or median (interquartile range), as appropriate, and categorical variables as frequencies and percentages.

Differences in eGFR across the five estimating equations were compared using the Kruskal–Wallis test. CKD-EPI 2009 was selected as the reference equation. Percent agreement, undertreatment, and overtreatment between each equation of interest and the reference equation were calculated. The corresponding 95% confidence intervals (CIs) were estimated simultaneously using the Goodman method for multinomial proportions. All statistical analyses were performed using STATA version 18.0 (StataCorp LLC., College Station, TX, USA). A two-sided *p*-value < 0.05 was considered statistically significant.

## 3. Results

### 3.1. Baseline Characteristics

A total of 46,788 participants with DM were included in the analysis (Figure 1). The mean age was 65.9 ± 12.9 years, 58.9% were female, and the mean body weight was 66.9 ± 15.2 kg. Overall, 65.8% of participants were receiving metformin therapy.

The median eGFR (mL/min/1.73 m^2^) calculated using different equations was as follows (Table 1): CKD-EPI 2021, 86.2 (IQR, 66.4–99.3); CKD-EPI 2009, 80.1 (IQR, 61.3–93.7); S-MDRD, 76.1 (IQR, 59.9–92.4); Thai GFR, 77.2 (IQR 63.5–90.8), and Cockcroft–Gault, 69.2 (IQR 50.1–93.4). The differences in eGFR across equations were statistically significant (*p*-value < 0.001).

### 3.2. CKD-EPI 2009 vs. 2021 Equations (Table 2)

The mean eGFR estimated using the CKD-EPI 2021 equation was 81.3 ± 24.7 mL/min/1.73 m^2^, compared with 76.4 ± 24.3 mL/min/1.73 m^2^ using the CKD-EPI 2009 equation, corresponding to a mean difference of +4.5 mL/min/1.73 m^2^ (*p*-value < 0.001). Overall, 8855 of 46,788 patients (19%) were reclassified to a one-stage higher eGFR category when the 2021 equation was applied. Notably, approximately one-third of patients classified as G3a or G3b according to the 2009 equation were reclassified to G2 or G3a, respectively, under the 2021 equation, reflecting an upward reclassification of CKD stages.

**Table 2:** Concordance of chronic kidney disease (CKD) stages between CKD-EPI 2021 and CKD-EPI 2009 equations. CKD stage was categorized solely based on eGFR.

CKD-EPI 2009: CKD Stages	CKD-EPI 2021: CKD Stages						
	G1	G2	G3a	G3b	G4	G5	Total
G1	15,248 (100.0%)	0	0	0	0	0	15,248 (32.6%)
G2	5331 (26.0%)	15,185 (74.0%)	0	0	0	0	20,516 (43.9%)
G3a	0	2164 (36.0%)	3849 (64.0%)	0	0	0	6013 (12.9%)
G3b	0	0	1060 (35.1%)	1961 (64.9%)	0	0	3021 (6.5%)
G4	0	0	0	257 (27.6%)	676 (72.4%)	0	933 (2.0%)
G5	0	0	0	0	43 (4.1%)	1014 (95.9%)	1057 (2.3%)
Total	20,579 (44.0%)	17,349 (37.1%)	4909 (10.5%)	2218 (4.7%)	719 (1.5%)	1014 (2.2%)	46,788

### 3.3. Participants with Impaired Kidney Function

Among participants with eGFR < 60 mL/min/1.73 m^2^, the median eGFRs were as follows: CKD-EPI 2021, 46.9 (IQR 34.8–54.1); CKD-EPI 2009, 46.5 (IQR 35.5–53.9); S-MDRD, 47.5 (IQR 37.2–54.4); Thai GFR, 50.0 (IQR 40.7–55.7); and Cockcroft–Gault, 44.7 (IQR 34.1–52.7) mL/min/1.73 m^2^.

Among participants with eGFR < 30 mL/min/1.73 m^2^, the median eGFRs were CKD-EPI 2021, 11.2 (IQR 6.6–22.7); CKD-EPI 2009, 12.9 (IQR 6.4–24.1); S-MDRD, 12.7 (IQR 6.6–23.8); Thai GFR, 15.3 (IQR 11.8–21.5); and Cockcroft–Gault, 21.2 (IQR 11.6–26.5) mL/min/1.73 m^2^.

### 3.4. Participants with Metformin Use (Table 3)

Among the 30,805 patients receiving metformin, the proportion classified as having eGFR < 30 mL/min/1.73 m^2^—suggesting potential inappropriate metformin use—was 1.1% using CKD-EPI 2021, 1.5% using CKD-EPI 2009, 1.4% using S-MDRD, 0.6% using the Thai GFR equation, and 3.7% using the Cockcroft–Gault equation.

**Table 3:** Concordance between metformin use and eGFR classification according to each estimating equation. CKD stage was categorized solely based on eGFR.

eGFR Equation	eGFR Category	No Metformin, No. (%)	Metformin Use, No. (%)
CKD-EPI 2021	<30 mL/min/1.73 m^2^	1393 (8.7%)	340 (1.1%)
	≥30 mL/min/1.73 m^2^	14,590 (91.3%)	30,465 (98.9%)
CKD-EPI 2009	<30 mL/min/1.73 m^2^	1532 (9.6%)	458 (1.5%)
	≥30 mL/min/1.73 m^2^	14,451 (90.4%)	30,347 (98.5%)
S-MDRD	<30 mL/min/1.73 m^2^	1489 (9.3%)	424 (1.4%)
	≥30 mL/min/1.73 m^2^	14,494 (90.7%)	30,381 (98.6%)
Thai GFR	<30 mL/min/1.73 m^2^	1136 (7.1%)	180 (0.6%)
	≥30 mL/min/1.73 m^2^	14,847 (92.9%)	30,625 (99.4%)
Cockcroft–Gault	<30 mL/min/1.73 m^2^	2114 (13.2%)	1140 (3.7%)
	≥30 mL/min/1.73 m^2^	13,869 (86.8%)	29,665 (96.3%)

### 3.5. CKD Classification Between eGFR Equations

Among the 11,024 participants with eGFR < 60 mL/min/1.73 m^2^ according to CKD-EPI 2009, concordant classification was observed in 98.3% using S-MDRD, 82.3% using the Thai GFR equation, and 91.9% using Cockcroft–Gault (Table 4).

Notably, none of the participants classified as having eGFR < 60 mL/min/1.73 m^2^ by CKD-EPI 2009 were reclassified to eGFR ≥ 90 mL/min/1.73 m^2^ by S-MDRD or the Thai GFR equation. However, 26 participants (0.24%) were reclassified to eGFR ≥ 90 mL/min/1.73 m^2^ by the Cockcroft–Gault equation. Among these 26 participants, the mean body weight was 117 kg, the mean age was 50 years, and 15 (57%) were male.

Among the 1990 participants with eGFR < 30 mL/min/1.73 m^2^ according to CKD-EPI 2009, concordant classification was observed in 94.5% using S-MDRD, 66.1% using the Thai GFR equation, and 92.8% using Cockcroft–Gault (Table 5).

### 3.6. Agreement of Metformin Use Between eGFR Equations

#### 3.6.1. eGFR Cutoff of 30 mL/Min/1.73 m^2^ (Eligibility Threshold) (Figure 2)

Using CKD-EPI 2009 as the reference, overall agreement was 99.70% (95% CI, 99.65–99.75%) for S-MDRD, 98.56% (95% CI, 98.45–98.66%) for the Thai GFR equation, 96.69% (95% CI, 96.53–96.84%) for Cockcroft–Gault, and 99.45% (95% CI, 99.39–99.52%) for CKD-EPI 2021.

Undertreatment occurred in 0.07% (95% CI, 0.04–0.09%) with S-MDRD, 0% with the Thai GFR equation, 3.01% (95% CI, 2.85–3.16%) with Cockcroft–Gault, and 0% with CKD-EPI 2021.

Overtreatment occurred in 0.23% (95% CI, 0.19–0.28%) with S-MDRD, 1.44% (95% CI, 1.33–1.55%) with the Thai GFR equation, 0.31% (95% CI, 0.26–0.36%) with Cockcroft–Gault, and 0.55% (95% CI, 0.48–0.62%) with CKD-EPI 2021.

**Figure 2:**
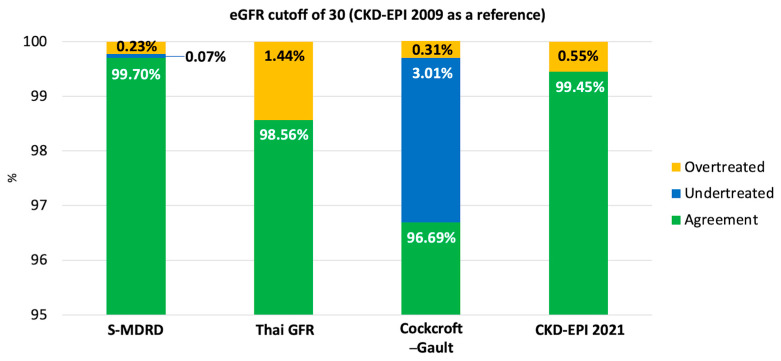
Agreement and potential undertreatment/over treatment using eGFR 30 mL/min/1.73 m^2^ by CKD-EPI 2009 as a reference.

#### 3.6.2. eGFR Cutoff of 45 mL/Min/1.73 m^2^ (Dose Adjustment Threshold) (Figure 3)

At the 45 mL/min/1.73 m^2^ threshold, overall agreement was 99.05% (95% CI, 98.97–99.14%) for S-MDRD, 96.09% (95% CI, 95.92–96.26%) for the Thai GFR equation, 89.60% (95% CI, 89.34–89.87%) for Cockcroft–Gault, and 97.73% (95% CI, 97.60–97.87%) for CKD-EPI 2021.

Undertreatment occurred in 0.45% (95% CI, 0.39–0.51%) with S-MDRD, 0% with the Thai GFR equation, 9.42% (95% CI, 9.15–9.68%) with Cockcroft–Gault, and 0% with CKD-EPI 2021.

Overtreatment occurred in 0.50% (95% CI, 0.43–0.56%) with S-MDRD, 3.91% (95% CI, 3.73–4.08%) with the Thai GFR equation, 0.98% (95% CI, 0.89–1.07%) with Cockcroft–Gault, and 2.27% (95% CI, 2.13–2.40%) with CKD-EPI 2021.

**Figure 3:**
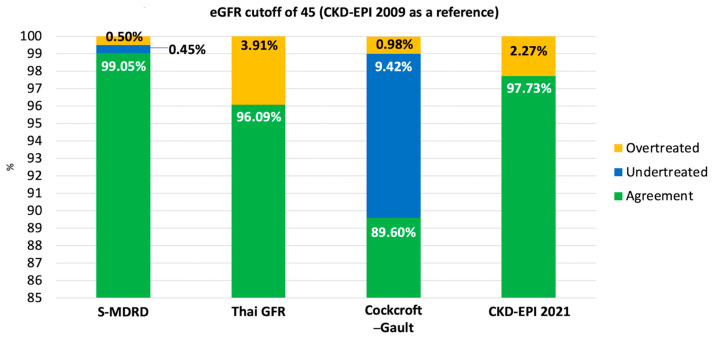
Agreement and potential undertreatment/over treatment using eGFR 45 mL/min/1.73 m^2^ by CKD-EPI 2009 as a reference.

## 4. Discussion

In this large cohort of 46,788 patients with DM and stable kidney function, we demonstrated that eGFR varies substantially depending on the equation used, leading to meaningful differences in CKD staging and metformin eligibility. CKD-EPI 2021 produced the highest eGFR values, followed by CKD-EPI 2009, Thai GFR, S-MDRD, and Cockcroft–Gault. Furthermore, switching from CKD-EPI 2009 to 2021 reclassified 19% of patients to a less severe CKD stage. Although most shifts occurred between adjacent categories, this degree of reclassification may influence CKD labeling, follow-up strategies, and medication dosing in routine clinical practice.

Among patients with reduced kidney function (eGFR < 60 mL/min/1.73 m^2^), the Thai GFR equation yielded higher eGFR estimates than CKD-EPI 2009, whereas Cockcroft–Gault produced the lowest values. In patients classified as eGFR < 30 mL/min/1.73 m^2^ by CKD-EPI 2009, upward reclassification above the 30 mL/min/1.73 m^2^ threshold occurred in 5% with S-MDRD, 7% with Cockcroft–Gault, and as high as 33% with Thai GFR. The tendency of the Thai GFR equation to yield higher values in advanced CKD raises concern for potential overtreatment when used to guide medication decisions, consistent with prior Thai studies demonstrating greater deviation from CKD-EPI at lower GFR levels [17].

Conversely, the lower eGFR estimates observed with Cockcroft–Gault may predispose to undertreatment. This likely reflects its reliance on body weight and lack of normalization to body surface area, which may introduce systematic variation—particularly in populations with diabetes, where overweight and obesity are common. In our cohort, use of Cockcroft–Gault would have resulted in undertreatment in approximately 3% of patients at the 30 mL/min/1.73 m^2^ eligibility threshold and more than 9% at the 45 mL/min/1.73 m^2^ dose-adjustment threshold.

Despite these differences, overall agreement for metformin eligibility across equations remained high (>98% at the 30 mL/min/1.73 m^2^ cutoff). However, even small discrepancies near treatment thresholds may carry important clinical implications. Emerging evidence suggests that discontinuing metformin solely on the basis of eGFR thresholds does not necessarily improve safety outcomes and may be associated with increased mortality or cardiovascular risk in advanced CKD [18,19]. Thus, balancing the prevention of rare adverse events such as metformin-associated lactic acidosis against the risk of unnecessarily withdrawing a cardioprotective therapy remains complex. However, as this study evaluated classification differences rather than clinical outcomes, the implications for safety or patient outcomes cannot be determined. Overall, our findings highlight that equation performance is stage-dependent, with the greatest variability observed in severe renal impairment, where clinical decisions are most sensitive to small changes in eGFR.

KDIGO currently recommends the CKD-EPI creatinine-based equation for estimating GFR. Our findings support the continued use of CKD-EPI-based equations for routine laboratory reporting. The modest upward shift observed with CKD-EPI 2021 is consistent with prior international reports and is likely attributable to removal of the race coefficient [20,21,22]. In predominantly non-Black populations, including Asian cohorts, this modification may increase eGFR estimates compared with the 2009 equation [15]. As laboratories transition to race-neutral equations, clinicians should be aware that apparent improvements in CKD stage may reflect recalibration of the estimating formula rather than true biological change. Clear communication during implementation is therefore essential to minimize misinterpretation and ensure appropriate clinical decision-making.

This study has several strengths. It includes a large, real-world cohort of patients with DM, reflecting routine clinical practice rather than highly selected trial populations. In addition, we performed a direct, head-to-head comparison of five commonly used creatinine-based eGFR equations within the same population, allowing meaningful evaluation of classification differences at clinically relevant treatment thresholds. However, several limitations should be acknowledged. First, measured GFR was not available; therefore, we cannot determine which equation most accurately reflects true kidney function. Our analysis addresses classification discordance rather than diagnostic accuracy. Second, the cross-sectional design limits causal inference and precludes the assessment of longitudinal outcomes and prescribing-related factors, including adverse drug reactions, metformin-associated complications, cardiovascular events, or mortality, and the underlying reasons for treatment selection or discontinuation. Third, CKD classification in this study was based solely on eGFR, as albuminuria data were not systematically available, which may have limited the identification of early-stage CKD. Further prospective studies, ideally including pragmatic trials or randomized controlled designs, are needed to determine whether equation-related differences in eGFR classification translate into meaningful differences in patient-centered outcomes. Such studies should assess the impact of equation selection on metformin continuation or discontinuation, adverse events, cardiovascular outcomes, and overall mortality, particularly among patients with advanced CKD where discrepancies are greatest.

## 5. Conclusions

Differences among eGFR equations lead to clinically relevant variation in CKD staging and metformin prescribing decisions. CKD-EPI 2009, CKD-EPI 2021, S-MDRD, and Thai GFR showed good overall agreement, whereas Cockcroft–Gault may underestimate renal function and potentially classify patients as ineligible for continued metformin use. CKD-EPI 2021 yielded slightly higher eGFR values than the 2009 equation in this predominantly Asian population, and stage shifts may reflect recalibration rather than true biological change. Until outcome data are available, CKD-EPI remains the preferred reference because of its consistency with other standard equations. Further prospective studies are needed to clarify the clinical impact of equation choice on metformin management and outcomes.

## Figures and Tables

**Table 1:** Prevalence of chronic kidney disease (CKD) stages according to different estimated glomerular filtration rate (eGFR) equations in patients with diabetes mellitus (n = 46,788).

CKD Stages						
N = 46,788	Stage 1 (eGFR ≥ 90)	Stage 2 (eGFR 60–89)	Stage 3a (eGFR 45–59)	Stage 3b (eGFR 30–44)	Stage 4 (eGFR 15–29)	Stage 5 (eGFR < 15)
CKD-EPI 2009						
No. (%)	15,248 (32.6%)	20,516 (43.9%)	6013 (12.9%)	3021 (6.5%)	933 (2.0%)	1057 (2.3%)
Median eGFR (IQR)	98.6 (94.0–105.6)	77 (69.1–84.1)	53.3 (49.4–56.7)	39.5 (35.5–42.4)	24.6 (20.5–27.6)	6.7 (4.9–9.3)
S-MDRD						
No. (%)	13,332 (28.5%)	21,694 (46.4%)	6772 (14.5%)	3077 (6.6%)	880 (1.9%)	1033 (2.2%)
Median eGFR (IQR)	103.1 (95.6–114.8)	75.1 (68.1–82.1)	53.5 (49.6–56.5)	39.8 (35.9–42.6)	24.6 (20.6–27.8)	6.9 (5.1–9.5)
Thai GFR						
No. (%)	12,302 (26.3%)	25,066 (53.6%)	6238 (13.3%)	1866 (4.0%)	688 (1.5%)	628 (1.3%)
Median eGFR (IQR)	100.8 (94.6–110.9)	75.6 (68.7–82.5)	54.1 (50.1–57.2)	39.9 (36.1–42.7)	21.2 (17.4–25.8)	11.6 (10.1–13.4)
Cockcroft–Gault						
No. (%)	13,010 (27.8%)	16,112 (34.4%)	8711 (18.6%)	5701 (12.2%)	2181 (4.7%)	1073 (2.3%)
Median eGFR (IQR)	112.1 (99.5–134.6)	73.1 (66.4–80.9)	52.8 (49.0–56.5)	38.6 (34.9–41.9)	25.2 (21.2–27.8)	9.0 (7.1–11.4)

Abbreviations: CKD = chronic kidney disease; eGFR = estimated glomerular filtration rate; CKD-EPI = Chronic Kidney Disease Epidemiology Collaboration; S-MDRD = Standard Modification of Diet in Renal Disease; Thai GFR = Thai glomerular filtration rate equation; IQR = interquartile range.

**Table 4:** Chronic kidney disease (CKD) staging among patients with eGFR < 60 mL/min/1.73 m^2^ (n = 11,024), using CKD-EPI 2009 as the reference equation. Median (IQR) eGFR values for this population were CKD-EPI 2009, 46.5 (35.5, 53.9); S-MDRD, 46.6 (36.31, 53.3); Thai GFR, 52.2 (42.9–58.0); and Cockcroft–Gault, 38.4 (28.2–48.5) mL/min/1.73 m^2^. CKD stage was categorized solely based on eGFR.

	CKD Stages by CKD-EPI 2009 (n = 11,024)					
	Stage 1 (eGFR ≥ 90)	Stage 2 (eGFR 60–89)	Stage 3a (eGFR 45–59)	Stage 3b (eGFR 30–44)	Stage 4 (eGFR 15–29)	Stage 5 (eGFR < 15)
CKD-EPI 2009 * No. (%)	0	0	6013 (54.5%)	3021 (27.4%)	933 (8.5%)	1057 (9.6%)
S-MDRD No. (%)	0	193 (1.8%)	5841 (53.0%)	3077 (27.9%)	880 (8.0%)	1033 (9.4%)
Thai GFR No. (%)	0	1952 (17.7%)	5890 (53.4%)	1866 (16.9%)	688 (6.2%)	628 (5.7%)
Cockcroft–Gault No. (%)	26 (0.2%)	870 (7.9%)	2774 (25.2%)	4156 (37.7%)	2125 (19.3%)	1073 (9.7%)

* Reference equation for cohort definition.

**Table 5:** Chronic kidney disease (CKD) staging among patients with eGFR < 30 mL/min/1.73 m^2^ (n = 1990), using CKD-EPI 2009 as the reference equation. Median (IQR) eGFR values for this population were CKD-EPI 2009, 12.9 (6.4–24.1); S-MDRD, 13.7 (6.8–24.9); Thai GFR, 21.84 (13.7–32.8); and Cockcroft–Gault, 13.9 (8.7–21.9) mL/min/1.73 m^2^. CKD stage was categorized solely based on eGFR.

	CKD Stages by CKD-EPI 2009 (n = 1990)					
	Stage 1 (eGFR ≥ 90)	Stage 2 (eGFR 60–89)	Stage 3a (eGFR 45–59)	Stage 3b (eGFR 30–44)	Stage 4 (eGFR 15–29)	Stage 5 (eGFR < 15)
CKD-EPI 2009 * No. (%)	0	0	0	0	933 (46.9%)	1057 (53.1%)
S-MDRD No. (%)	0	0	0	109 (5.5%)	848 (42.6%)	1033 (51.9%)
Thai GFR No. (%)	0	0	0	674 (33.9%)	688 (34.6%)	628 (31.5%)
Cockcroft–Gault No. (%)	0	0	5 (0.3%)	138 (6.9%)	776 (39.0%)	1071 (53.8%)

* Reference equation for cohort definition.

## Data Availability

Data available on request due to ethical restrictions.

## Abbreviations

The following abbreviations are used in this manuscript:

CKD	Chronic Kidney Disease
CKD-EPI	Chronic Kidney Disease Epidemiology Collaboration
DM	Diabetes Mellitus
eGFR	Estimated Glomerular Filtration Rate
GFR	Glomerular Filtration Rate
KDIGO	Kidney Disease: Improving Global Outcomes
MDRD	Modification of Diet in Renal Disease
S-MDRD	Standard Modification of Diet in Renal Disease
